# Mutations in the DNA polymerase binding pathway affect the immune microenvironment of patients with small‐cell lung cancer and enhance the efficacy of platinum‐based chemotherapy

**DOI:** 10.1002/cai2.84

**Published:** 2023-07-11

**Authors:** Anqi Lin, Weiming Mou, Lingxuan Zhu, Tao Yang, Chaozheng Zhou, Jian Zhang, Peng Luo

**Affiliations:** ^1^ Department of Oncology, Zhujiang Hospital Southern Medical University Guangzhou Guangdong China; ^2^ The First Clinical Medical School Southern Medical University Guangzhou Guangdong China; ^3^ Department of Urology, Shanghai General Hospital Shanghai Jiao Tong University School of Medicine Shanghai China; ^4^ Department of Etiology and Carcinogenesis National Cancer Center, National Clinical Research Center for Cancer, Cancer Hospital, Chinese Academy of Medical Sciences and Peking Union Medical College Beijing China; ^5^ Department of Medical Oncology National Cancer Center, National Clinical Research Center for Cancer, Cancer Hospital, Chinese Academy of Medical Sciences and Peking Union Medical College Beijing China

**Keywords:** chemotherapy resistance, platinum, prognosis, small‐cell lung cancer, tumor microenvironment

## Abstract

**Background:**

Small‐cell lung cancer (SCLC) is characterized by its high malignancy and is associated with a poor prognosis. In the early stages of the disease, platinum‐based chemotherapy is the recommended first‐line treatment and has demonstrated efficacy. However, SCLC is prone to recurrence and is generally resistant to chemotherapy in its later stages.

**Methods:**

Here, we collected samples from SCLC patients who received platinum‐based chemotherapy, performed genomic and transcriptomic analyses, and validated our results with publicly available data.

**Results:**

SCLC patients with DNA polymerase binding pathway mutations had an improved prognosis after platinum chemotherapy compared with patients without such mutations. Patients in the mutant (MT) group had higher infiltration of T cells, B cells, and M1 macrophages compared with patients without DNA polymerase binding pathway mutations.

**Conclusions:**

DNA polymerase binding pathway mutations can be used as prognostic markers for platinum‐based chemotherapy in SCLC.

## Graphical abstract

Our study demonstrates that mutation in the DNA polymerase binding pathway can serve as a prognostic marker for platinum‐based chemotherapy in small‐cell lung cancer (SCLC). Furthermore, we identified the possible mechanisms by which such mutation could enhance antitumor immunity and sensitivity to chemotherapy. Our findings provide important insights into the design of therapeutic and clinical trials aimed at improving outcomes for SCLC patients and those with other DNA polymerase binding pathway‐associated tumors.

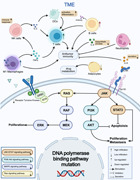

**Abbreviations**
DCs: dendritic cells
DFS: disease‐free survival
ERK: extraneous signal‐regulated kinase
GO: Gene Ontology
GSEA: Gene Set Enrichment Analysis
IL: interleukin
IRBs: Institutional Review Boards
KEGG: Kyoto Encyclopedia of Genes and Genomes
KM: Kaplan‐Meier
MAPK: mitogen‐activated protein kinase
MSigDB: Molecular Signature Database
MT: mutant
NK: natural killer
OS: overall survival
PCA: principal component analysis
PI3K: phosphatidylinositol 3‐kinase
SCLC: small‐cell lung cancer
ssGSEA: single sample gene set enrichment analysis
TILs: tumor infiltrating lymphocytes
TME: tumor microenvironment
WT: wild‐type

## 1 INTRODUCTION

Lung cancer is the most prevalent malignant neoplasm, with an annual incidence of around 1.8 million cases worldwide. This disease is a considerable health threat, as it has the highest death rate of all malignant cancers and causes about 1.6 million deaths each year [1, 2]. Approximately 15% of lung cancer cases are attributed to small‐cell lung cancer (SCLC), a subtype that is classified as a high‐grade neuroendocrine tumor. Chemotherapy is highly effective for treating SCLC, with the classic first‐line regimen, platinum‐based chemotherapy, initially achieving a 50%–75% response rate [3, 4]. However, SCLC is generally drug‐resistant at later stages, recurrence is almost inevitable, and the efficacy of late‐stage treatment is poor [5]. Currently, patients with limited‐stage SCLC have a 5‐year survival rate of 20%–25%, while this rate is just 2% for those with extensive‐stage disease [6, 7]. The identification of predictive markers for the effectiveness of platinum‐based chemotherapy is crucial for enhancing the overall prognosis of SCLC patients. Previous research conducted by our team demonstrated that mutations in certain genes are linked to the prognosis of SCLC patients who underwent platinum‐based chemotherapy. Despite existing research on the subject, there remains a lack of systemic pathway analyses regarding the factors that influence the prognosis of SCLC patients who receive platinum‐based chemotherapy [8].

The tumor microenvironment (TME) is closely linked to chemotherapeutic drug resistance, and the local TME consisting of immune cells with different phenotypes and functions can strongly impact the response to chemotherapy [9]. Antitumor immunity has been shown to reverse chemoresistance in tumors by increasing the rate of cancer cell apoptosis [10]. Additionally, the TME can affect the sensitivity of SCLC to chemotherapeutic agents, which can enhance the sensitivity of tumor cells to immune cell‐mediated killing [11]. In breast cancer, a higher degree of infiltration of tumor‐infiltrating lymphocytes (TILs) has been linked to increased susceptibility to chemotherapy regimens that feature anthracycline [12]. Chemokines also play important roles in the acquisition of chemoresistance [13, 14]. Numerous immune cell types within the TME secrete chemokines to facilitate intercellular signaling, resulting in the activation of several downstream pathways, including but not limited to nuclear factor Kappa B, the Janus kinase signal transducer and activator of transcription (JAK/STAT), and Notch signaling, all of which can promote tumor growth [13]. Higher C‐X‐C motif chemokine ligand 2 (CXCL2) expression levels have been found in patients with platinum‐resistant ovarian cancer [15], and CXCL2 has been shown to increase cisplatin resistance [14].

DNA polymerase plays a key role in resistance to platinum‐based drugs [16]. Many chemotherapeutic agents prevent tumor cell division by inducing DNA damage [17]. DNA polymerase effectively binds damaged nucleotides during translesion DNA synthesis, which reduces the efficacy of chemotherapeutic agents and contributes to tumor cell survival [16]. In numerous cancer types, specific mutations and polymorphisms in genes encoding DNA polymerases have been identified as supplementary prognostic indicators [18, 19, 20]. In certain cell lines, defects in DNA polymerases have been shown to enhance sensitivity to cisplatin or oxaliplatin [21]. DNA polymerases are also affected by the TME, which in turn affects the DNA repair process and alters tumor progression [22]. Disruption of DNA polymerase may simultaneously increase susceptibility to genotoxins and induce an innate immune response [23]. Many genes in the DNA polymerase binding pathway (GO: 0070182) have been found to be closely linked to chemoresistance and TME [24, 25, 26]. However, the relationship between mutations in the DNA polymerase binding pathway and chemoresistance in SCLC is not well understood.

In this study, we investigated the influence of mutations in the DNA polymerase binding pathway on chemotherapy resistance in SCLC patients. We utilized genomic and transcriptomic data collected from SCLC patients who received platinum‐based drug treatment at three major tertiary hospitals located in Guangdong province, which we refer to as the “Local‐SCLC” cohort. We validated our findings using previously published data from SCLC patients treated with platinum‐based drugs (the “George‐SCLC” cohort) [27]. We found that mutations in the DNA polymerase binding pathway can be used as prognostic markers in platinum‐treated SCLC patients. The presence of these mutations may improve the prognosis of these patients by affecting the TME and inhibiting chemoresistance‐associated pathways.

## 2 METHODS

### 2.1 Data sources

To assess the relationship between DNA polymerase binding pathway mutation status and chemotherapy resistance in SCLC, we analyzed two SCLC cohorts treated with platinum‐based drugs. The genomes and transcriptomes of SCLC samples obtained from the “Local‐SCLC” cohort, consisting of patients from three large tertiary hospitals located in Guangdong province, were sequenced. We also collected clinical information, such as the TNM classification of malignant tumor (TNM) stage, sex, smoking history, and age. The Institutional Review Boards (IRBs) approved the study. Detailed sample processing methods can be found in Lin et al.'s study [28]. In addition to our Local‐SCLC cohort, we downloaded genomic, transcriptomic, and clinical data from the previously published George‐SCLC cohort [27]. Our Local‐SCLC cohort contained 45 patients and the George‐SCLC cohort contained 68 patients.

### 2.2 Relationships between DNA polymerase pathway mutations and chemotherapy resistance

**Figure 1:**
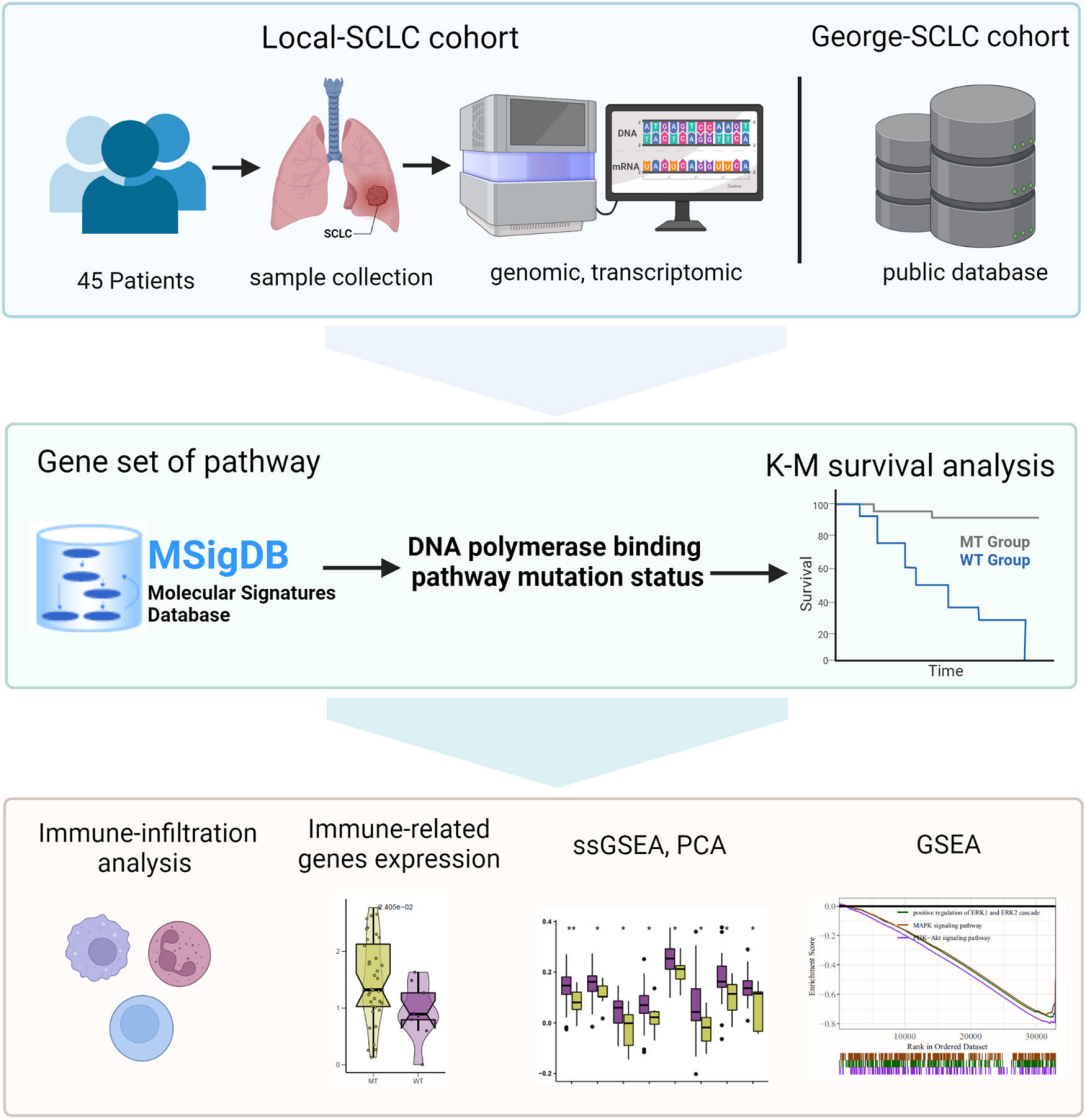
Workflow of our study. GSEA, gene set enrichment analysis; KM, Kaplan‐Meier, PCA, principal component analysis; SCLC, small‐cell lung cancer; ssGSEA, single sample gene set enrichment analysis.

The DNA polymerase binding pathway was obtained from the Gene Ontology (GO) database [29, 30]. The gene set was downloaded from the Molecular Signature Database (MSigDB) [31]. A mutation occurring in one or more genes that are part of the DNA polymerase binding pathway within the genome of SCLC samples was referred to as a pathway mutation. We divided SCLC patients into mutant (MT) and wild‐type (WT) groups according to the mutation status of the DNA polymerase binding pathway. Kaplan‐Meier (KM) survival analysis was performed for the Local‐SCLC and George‐SCLC cohorts. Figure 1 and Graphical Abstract Image were created with Biorender (BioRender.com).

### 2.3 Immune profiling analysis

We used the microenvironment cell populations (MCP)‐counter, estimating the proportions of immune and cancer cells (EPIC), quantiseq, CIBERSORT, and xCell algorithms from the immuno‐oncology biological research (IOBR) package for immune‐infiltration analysis [32]. These algorithms utilized messenger RNA (mRNA) gene expression data from the Local‐SCLC and George‐SCLC cohorts to quantify the absolute abundance of immune and stromal cells in tumor tissues. To evaluate the expression profile of immune‐related genes in SCLC, we utilized the genes previously identified as relevant to the immune process [33, 34].

### 2.4 Pathway enrichment analysis

The degree of immune‐related pathway activation in SCLC patient samples was assessed using principal component analysis (PCA) for the immune‐related gene set included in the IOBR package [32]. The SCLC mRNA data were analyzed by single‐sample gene set enrichment analysis (ssGSEA) using the gene set variation analysis package [35]. Differential analysis of mRNA data from the MT and WT groups was performed using the limma package, and the differentially expressed genes were ranked according to log fold change (FC). Gene set enrichment analysis (GSEA) was performed using the ClusterProfiler package [36]. The ssGSEA and GSEA analysis entries were obtained from GO [29, 30], Kyoto Encyclopedia of Genes and Genomes (KEGG) [37], and Reactome [38]. The gene sets for these pathways were downloaded from MSigDB [31]. *p* Values less than 0.05 were considered statistically significant.

### 2.5 Statistical analysis

The statistical analyses were conducted using R software (version 4.1.2). For comparing two continuous variables, the Mann‐Whitney *U* test was applied, while Fisher's exact test was utilized to compare two categorical variables. We used the KM method to perform survival analyses and calculate log‐rank *p* values. A significance level of 0.05 was chosen for all analyses, and *p* values were two‐tailed.

## 3 RESULTS

### 3.1 DNA polymerase binding pathway mutations are prognostic markers for SCLC patients receiving platinum‐based chemotherapy

**Figure 2:**
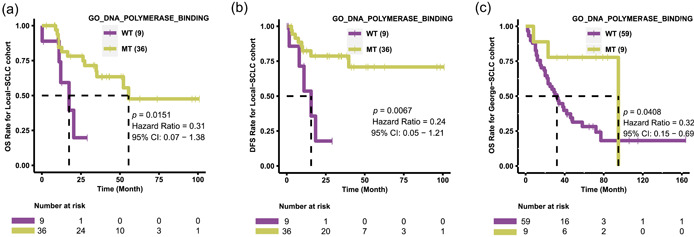
SCLC patients with mutations in the DNA polymerase binding pathway have a better prognosis after platinum‐based therapy. (a) KM survival analysis (OS) for the MT and WT groups in the Local‐SCLC cohort; (b) KM survival analysis (DFS) for the MT and WT groups in the Local‐SCLC cohort; (c) KM survival analysis (OS) for the MT and WT groups in the George‐SCLC cohort. DFS, disease‐free survival; KM, Kaplan‐Meier; MT, mutant; OS, overall survival; SCLC, small‐cell lung cancer; WT, wild‐type.

A diagram of our workflow is shown in Figure 1. There were 36 patients in the MT group and 9 patients in the WT group of the Local‐SCLC cohort. There were no significant differences in sex (*p* = 1), age (*p* = 0.071), TNM stage (*p* = 0.159), alcohol consumption (drinking, *p* = 0.261), smoking history (*p* = 0.402), or cigarette consumption (pack‐years, *p* = 0.333) between the WT and MT groups (Table 1). We then performed KM survival analysis and found that patients in the MT group of the Local‐SCLC cohort had a better prognosis (overall survival [OS]: *p* = 0.0151, hazard ratio [HR] = 0.31, 95% confidence interval [CI]: 0.07–1.38) (Figure 2a); disease‐free survival (DFS): *p* = 0.0067, HR = 0.24, 95% CI: 0.07–1.38, Figure 2b), while the WT group exhibited resistance to platinum drugs. We then analyzed the George‐SCLC cohort and found no significant difference in age (*p* = 0.370), TNM stage (*p* = 0.211), or cigarette consumption (pack‐years, *p* = 0.589) between the WT and MT groups (Table 2). Consistent with our findings from the Local‐SCLC cohort, patients in the MT group of the George‐SCLC cohort had a better prognosis than those in the WT group (OS: *p* = 0.0408, HR = 0.32, 95% CI: 0.15–0.69, Figure 2c). We analyzed the mutation profiles in both the MT and WT groups and found that the MT group had a significantly higher frequency of co‐occurrence genes compared with the WT group (Supporting Information: Figure 1).

**Table 1:** Clinical characteristics of patients in the Local‐SCLC cohort.

	DNA polymerase binding‐MT (*n* = 36)	DNA polymerase binding‐WT (*n* = 9)	Overall (*n* = 45)	*p *Value
Sex, *n* (%)				1
Female	7 (19.4)	1 (11.1)	8 (17.8)	
Male	29 (80.6)	8 (88.9)	37 (82.2)	
TNM_stage, *n* (%)				0.159
I	15 (41.7)	1 (11.1)	16 (35.6)	
II	6 (16.7)	1 (11.1)	7 (15.6)	
III	13 (36.1)	7 (77.8)	20 (44.4)	
IV	1 (2.8)	0 (0.0)	1 (2.2)	
Missing	1 (2.8)	0 (0.0)	1 (2.2)	
Smoking, *n* (%)				0.402
Nonsmoker	7 (19.4)	3 (33.3)	10 (22.2)	
Smoker	28 (77.8)	6 (66.7)	34 (75.6)	
Missing	1 (2.8)	0 (0.0)	1 (2.2)	
Drinking, *n* (%)				0.261
Drinker	21 (58.3)	3 (33.3)	24 (53.3)	
Nondrinker	14 (38.9)	6 (66.7)	20 (44.4)	
Missing	1 (2.8)	0 (0.0)	1 (2.2)	
Age (years)				0.071
Mean (SD)	59.2 (11.0)	66.6 (6.86)	60.7 (10.7)	
Median [Min, Max]	60.5 [23.0, 76.0]	64.0 [56.0, 74.0]	63.0 [23.0, 76.0]	
Pack‐years				0.333
Mean (SD)	39.3 (34.8)	30.0 (36.7)	37.4 (35.0)	
Median [Min, Max]	40.0 [0, 150]	20.0 [0, 100]	33.8 [0, 150]	
Missing, *n* (%)	1 (2.8)	0 (0.0)	1 (2.2)	

Abbreviations: MT, mutant; SCLC, small‐cell lung cancer; TNM, the TNM classification of malignant tumor; WT, wild‐type.

**Table 2:** Clinical characteristics of patients in the George‐SCLC cohort.

	DNA polymerase binding‐MT (*n *= 9)	DNA polymerase binding‐WT (*n* = 59)	Overall (*n* = 68)	*p* Value
TNM stage, *n* (%)				0.211
I	4 (44.4)	23 (39.0)	27 (39.7)	
II	4 (44.4)	10 (16.9)	14 (20.6)	
III	1 (11.1)	18 (30.5)	19 (27.9)	
IV	0 (0.0)	8 (13.6)	8 (11.8)	
Age (years)				0.370
Mean (SD)	67.1 (9.39)	64.1 (8.81)	64.5 (8.87)	
Median [Min, Max]	70.0 [51.0, 81.0]	64.0 [47.0, 83.0]	64.0[47.0, 83.0]	
Pack‐years				0.589
Mean (SD)	39.0 (25.8)	45.6 (25.7)	44.4 (25.5)	
Median [Min, Max]	37.5 [0, 70.0]	45.0 [0, 100]	45.0 [0, 100]	
Missing, *n* (%)	1 (11.1)	20 (33.9)	21 (30.9)	

Abbreviations: MT, mutant; SCLC, small‐cell lung cancer; TNM, the TNM classification of malignant tumor; WT, wild‐type.

### 3.2 DNA polymerase binding pathway mutations can affect the TME of SCLC

**Figure 3:**
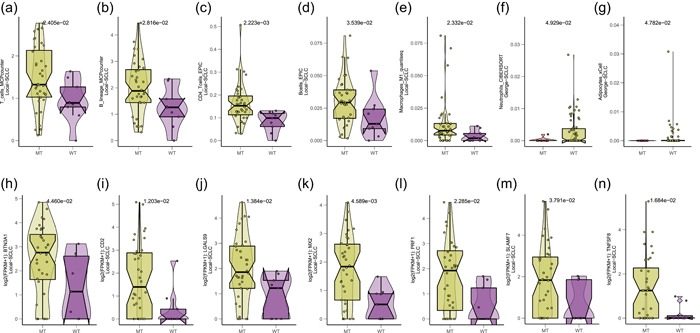
Effects of DNA polymerase binding pathway mutations on the tumor microenvironment of SCLC tumors. (a–e) Immune and stromal cell infiltration in the Local‐SCLC cohort; (f, g) immune cell and stromal cell infiltration in the George‐SCLC cohort; (h–n) immune‐related gene expression in the Local‐SCLC cohort. MT, mutant; SCLC, small‐cell lung cancer; WT, wild‐type.

To explore the effects of the DNA polymerase binding pathway on the TME of SCLC, we integrated multiple analyses of immune cell infiltration. The MCP‐counter, EPIC, quantiseq, CIBERSORT, and xCell algorithms are reliable methods for accurately estimating the absolute abundance of immune and stromal cell populations in SCLC using transcriptomic data. The results of these analyses showed that in the Local‐SCLC cohort, the infiltration of B cells, T cells, and M1 macrophages was higher in the MT group than in the WT group (Figure 3a–e). We also observed increased expression levels of immune‐related genes, such as *SLAMF7*, *TNFSF8*, *BTN3A1*, *CD2*, *LGALS9*, *PRF1*, and *MX2*, in tumor samples from the MT group of the Local‐SCLC cohort (Figure 3h–n). Additionally, in the George‐SCLC cohort, we found increased neutrophil and adipocyte infiltration in the TME of patients in the WT group (Figure 3f,g).

### 3.3 DNA polymerase binding pathway mutations can affect other signaling pathways in SCLC

**Figure 4:**
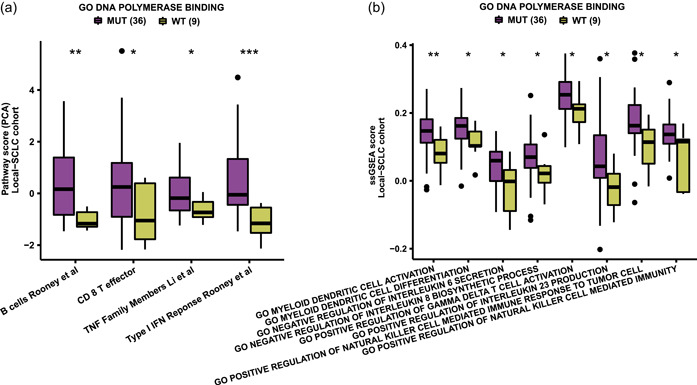
DNA polymerase binding pathway mutations can regulate signaling pathway activation in SCLC. (a) PCA results in the Local‐SCLC cohort; (b) ssGSEA results in the Local‐SCLC cohort. PCA, principal component analysis; GO, Gene Ontology; SCLC, small‐cell lung cancer; ssGSEA, single sample gene set enrichment analysis; WT, wild‐type.

**Figure 5:**
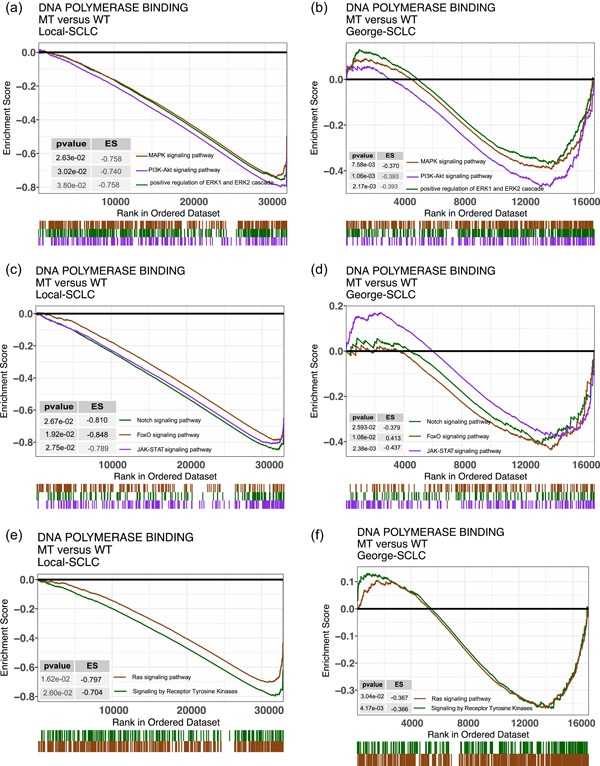
Pathways related to DNA polymerase binding pathway mutations in SCLC patients. (a, c, e) GSEA results in the Local‐SCLC cohort; (b, d, f) GSEA results in the George‐SCLC cohort. GSEA, gene set enrichment analysis; MT, mutant; SCLC, small‐cell lung cancer; WT, wild‐type.

To further understand the impact of DNA polymerase binding pathway mutations on chemoresistance in SCLC, we explored the extent of pathway activation in tumors using PCA, ssGSEA, and GSEA. We found that the following pathways were highly activated in the MT group of the Local‐SCLC cohort: B cells, CD8+ effector T cells, myeloid dendritic cell activation, gamma delta (γδ) T cell activation, natural killer (NK) cell‐mediated immunity, and other pathways related to immune cell function (Figure 4a,b). These findings are consistent with a heightened infiltration of immune cells, such as T and B cells, in the TME. In addition, some pathways regulating the production and secretion of cytokines, including interleukin (IL)‐6 and IL‐8, were significantly different between the MT and WT groups of the Local‐SCLC cohort (Figure 4b). GSEA analysis showed that MAPK, Notch, PI3K‐Akt, FoxO, JAK‐STAT, and ERK1/2 signaling were significantly downregulated in the MT group of the Local‐SCLC cohort (Figure 5a,b). These pathways are closely associated with chemoresistance, tumor progression, and metastasis. The GSEA results for the George‐SCLC cohort were consistent with those of the Local‐SCLC cohort (Figure 5c,d).

## 4 DISCUSSION

In the present study, we aimed to explore the specific pathways associated with the improved prognosis of SCLC patients treated with platinum‐based chemotherapy. We found a significant association between mutations in the DNA polymerase binding pathway and improved prognosis in SCLC patients receiving platinum‐based chemotherapy. This finding was validated in the George‐SCLC cohort. We then analyzed the TME and activation of cancer‐associated pathways in SCLC to explore the potential mechanisms by which DNA polymerase binding pathway mutations can confer sensitivity to platinum‐based chemotherapy. Our data suggested that patients in the MT group had a greater infiltration of immune cells involved in antitumor immunity, such as T cells and M1 macrophages [39, 40, 41, 42]. In contrast, the WT group had a greater infiltration of cells associated with platinum chemoresistance or immune evasion, such as neutrophils and adipocytes [43, 44]. We also found that many pathways that are strongly associated with chemoresistance, tumor progression, and metastasis were downregulated in the MT group compared with the WT group. Overall, these data suggest an association between DNA polymerase binding pathway mutations and improved SCLC patient survival after platinum‐based chemotherapy.

Tumor development is highly dependent on the specific TME, which has important prognostic implications and can affect sensitivity to chemotherapeutic agents [45, 46]. NK cells, M1 macrophages, cytotoxic lymphocytes, and B cells are associated with a better prognosis in many cancers [39, 47, 48]. NK cells secrete the cytokine IL‐2 in the TME, which induces dendritic cell activation and promotes T cell proliferation and differentiation [49, 50]. Dendritic cells are a key determinant in the initiation and maintenance of an effective T cell‐mediated antitumor immune response, and CD8+ T cells are activated by dendritic cell‐mediated antigen presentation [51, 52]. M1 macrophages are considered to have an anticancer phenotype and can promote antitumor immunity [53]. M1 macrophage activation is characterized by high IL‐23 expression levels, and IL‐23 activation allows macrophages to produce tumor necrosis factor (TNF)‐α, an important cytokine for immunogenic tumor cell killing by CD8 + T cells and NK cells [54, 55]. Increased M1 macrophage infiltration in the TME is indicative of a better prognosis [56]. Activated CD8+ and γδ‐T cells are typically involved in type I immune responses and have been linked to a more favorable prognosis in patients with lung cancer [57, 58]. Tumor‐associated B cells in the lung can differentiate into plasma cells, which produce antibodies that specifically recognize tumor‐associated antigens. The presence of B cells has been associated with better long‐term survival in lung cancer patients, suggesting a protective role for B cells and antibodies in antitumor immunity [59]. B cells not only produce antibodies but also enhance tumor‐associated T cell responses by promoting T cell activation and expansion. Previous research has suggested that high levels of T cell and B cell coinfiltration are associated with prolonged survival in lung cancer patients [60]. The mutation status of the DNA polymerase binding pathway is closely linked to the immune microenvironment and may hold promising predictive value in assessing the efficacy of tumor immunotherapy. However, further investigations are necessary to comprehensively elucidate the association between mutations in the DNA polymerase binding pathway and the response to immunotherapy.

Additionally, certain cell types and immune factors associated with chemoresistance or tumor cell proliferation were decreased in the TME of patients in the MT group. It has been shown that tumor‐associated neutrophils can promote lung cancer growth, participate in the establishment of an immunosuppressive TME, and support metastasis [43, 61, 62]. Adipocytes can reduce the cytotoxicity of chemotherapeutic drugs in the TME through uptake and metabolism, leading to chemoresistance [44]. IL‐8 is a member of the CXC chemokine family that is involved in lung cancer progression and metastasis by regulating angiogenesis, cancer cell survival and growth, tumor cell motility, leukocyte infiltration, and modifying immune responses in both the tumor and TME [63]. In cisplatin therapy, increased IL‐6 secretion leads to tumor cell migration and proliferation, promotes lung cancer metastasis, and is associated with poor prognosis [64, 65, 66].

The expression of immune‐related genes can improve the prognosis of patients treated with platinum chemotherapy by affecting the TME. Our analysis of immune‐related genes showed that the expression levels of some genes were significantly higher in the MT group than in the WT group, including *SLAMF7*, *TNFSF8*, *BTN3A1*, *PRF1*, *MX2*, and *LGALS9*. *SLAMF7* is involved in the cytotoxicity of activated NK cells, and *SLAMF7* expression in primary tumors is associated with an improved response to chemotherapy [67, 68, 69, 70]. Additionally, *TNFSF8* can promote T cell proliferation, and high *TNFSF8* expression levels are associated with a better response to chemotherapy [71, 72]. *BTN3A1* is involved in the activation of γδ‐T cells and promotes antitumor immunity [73]. *PRF1* is highly expressed in TILs, and its expression is associated with sensitivity to chemotherapy [12]. *MX2* is associated with TIL infiltration, and patients whose tumors had higher *MX2* expression levels had better prognoses [74]. *CD2* expression in lymphocytes can induce apoptosis, promote immune activation, inhibit tumor cell proliferation, and support tumor regression [75]. The immune‐related gene *LGALS9* can also inhibit tumor cell proliferation by inducing apoptosis [76, 77].

Many pathways that are strongly associated with chemoresistance and tumor progression and metastasis were downregulated in the MT group relative to the WT group. MAPK signaling plays a key role in patient outcomes and sensitivity to anticancer therapies [78]. ERK, a member of the MAPK family, is closely associated with drug resistance in lung cancer. Aberrant ERK activation is observed in drug‐resistant cells from a variety of cancers, including lung cancer [79, 80]. The Notch signaling pathway is typically activated in numerous cancers, and its activation promotes cancer progression. Inhibition of Notch can restrain tumor growth and sensitize cancer cells to a variety of chemotherapeutic agents [81]. PI3Ks are also thought to contribute to chemoresistance. Activation of the PI3K/AKT pathway leads to the phosphorylation of a cascade of proteins, promoting tumor cell proliferation, inhibiting apoptosis, facilitating invasion and metastasis, and regulating endothelial cell growth and angiogenesis [82]. Activated PI3K/AKT signaling leads to chemoresistance in the TME by inhibiting immune responses and activating the survival signaling pathway [83]. Furthermore, activation of *FOXO1* or *FOXO3a*, members of the FOXO family, can lead to chemoresistance in certain cancers. The expression and activation of *FOXO1* in esophageal squamous cell carcinoma induce TGFβ1 expression and make these cancer cells significantly resistant to chemotherapeutic agents [84]. Upon exposure to cisplatin, *FOXO1* was activated in gastric cancer cells. The upregulation and activation of *FOXO1* could trigger the PI3K/AKT pathway, leading to the inhibition of cisplatin‐induced apoptosis [85]. The IL‐6/JAK/STAT3 signaling pathway is frequently overactivated in various cancer types, and its dysregulation is commonly correlated with unfavorable clinical outcomes [86].

## 5 CONCLUSIONS

In this study, we found that mutations in the DNA polymerase binding pathway can be used as prognostic markers for platinum‐based chemotherapy in SCLC. These mutations are associated with improved survival following platinum‐based chemotherapy in SCLC. We explored the possible mechanisms by which mutations in the DNA polymerase binding pathway could affect the TME, promote antitumor immunity, and increase tumor sensitivity to chemotherapeutic agents. Our data showed that mutations in this pathway could inhibit pathways associated with chemoresistance, tumor progression, and metastasis. In addition, the present study provides important evidence for the design of therapeutic and clinical trials for SCLC and other tumor types associated with DNA polymerase binding pathway mutations.

## AUTHOR CONTRIBUTIONS

**Anqi Lin**: Data curation (equal); formal analysis (equal); investigation (equal); resources (equal); software (equal); validation (equal); visualization (equal); writing—original draft (equal); writing—review and editing (equal). **Weiming Mou**: Data curation (equal); formal analysis (equal); investigation (equal); resources (equal); software (equal); validation (equal); visualization (equal); writing—original draft (equal); writing—review and editing (equal). **Lingxuan Zhu**: Investigation (equal); writing—review and editing (equal). **Tao yang**: investigation (equal); writing—review and editing (equal). **Chaozheng Zhou**: Investigation (equal); writing—review and editing (equal). **Jian Zhang**: Conceptualization (equal); funding acquisition (equal); project administration (equal); resources (equal); supervision (equal); writing—review and editing (equal). **Peng Luo**: Conceptualization (equal); funding acquisition (equal); project administration (equal); resources (equal); supervision (equal); writing—review and editing (equal).

## CONFLICT OF INTEREST STATEMENT

The authors declare no conflict of interest.

## ETHICS STATEMENT

The research presented here was performed in accordance with the Declaration of Helsinki and has been approved by the ethics committee of the Zhujiang Hospital of Southern Medical University (2019‐KY‐051‐02).

## INFORMED CONSENT

The patients/participants provided their written informed consent to participate in this study.

## Supporting information

Figurementary Figure 1.

## Data Availability

All the data generated or analyzed during this study are included in this published article (https://www.cbioportal.org/study/summary?id=sclc_ucologne_2015). All other relevant data are available from the authors of this study upon request.
