# Supplementary figures and images for: Mutations in the DNA polymerase binding pathway affect the immune microenvironment of patients with small‐cell lung cancer and enhance the efficacy of platinum‐based chemotherapy
Source: Cancer Innov. 2023 Jul 11;2(6):500–12. doi: 10.1002/cai2.84 (PMC10730006; doi:10.1002/cai2.84)

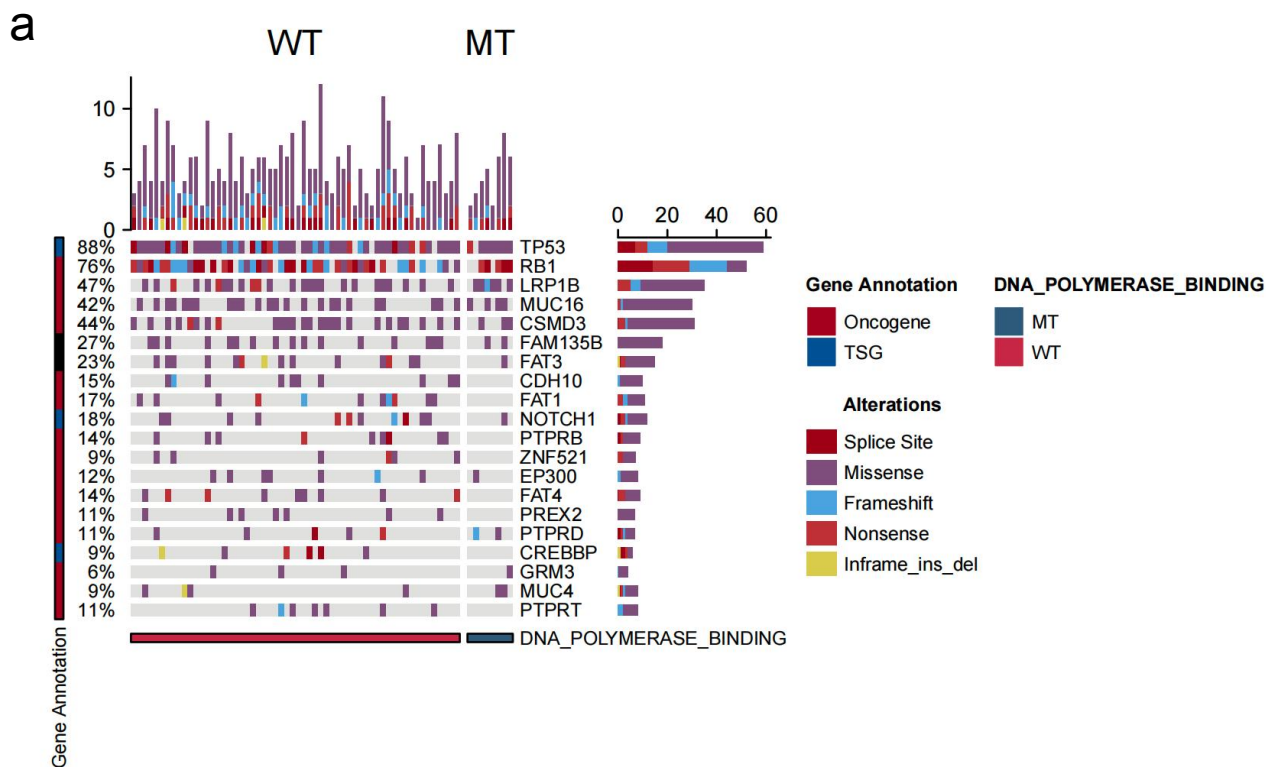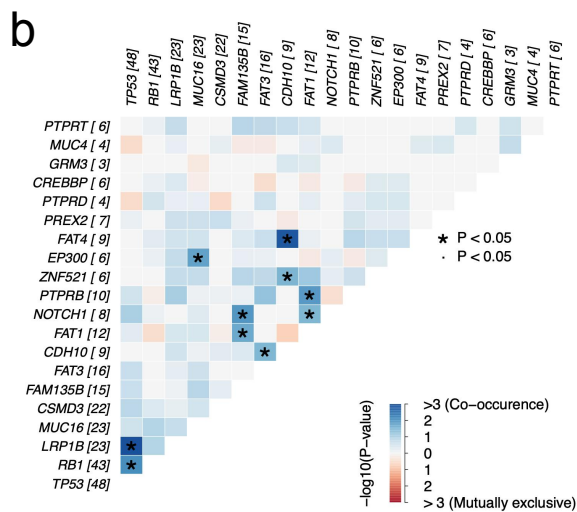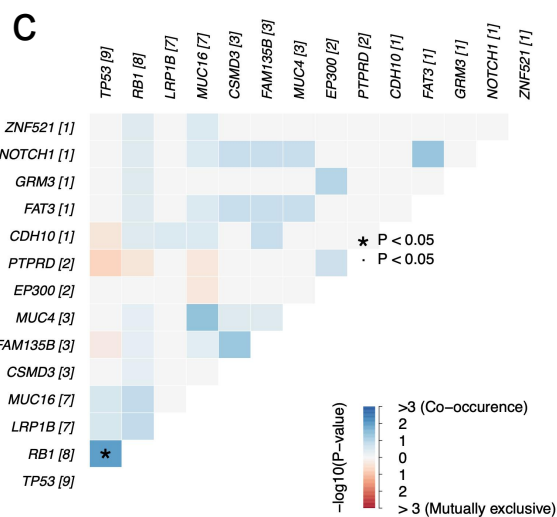

Supplement: Supplementary file 1 — Figurementary Figure 1. [file CAI2-2-500-s001.pdf]
